# Domain Cell Theory supports the independent evolution of the Eukarya, Bacteria and Archaea and the Nuclear Compartment Commonality hypothesis

**DOI:** 10.1098/rsob.170041

**Published:** 2017-06-28

**Authors:** James T. Staley

**Affiliations:** Department of Microbiology and Astrobiology Program, University of Washington, Seattle, WA 98195, USA

**Keywords:** evolution, cell theory, Tree of Life

## Abstract

In 2015, the Royal Society of London held a meeting to discuss the various hypotheses regarding the origin of the Eukarya. Although not all participants supported a hypothesis, the proposals that did fit into two broad categories: one group favoured ‘Prokaryotes First’ hypotheses and another addressed ‘Eukaryotes First’ hypotheses. Those who proposed Prokaryotes First hypotheses advocated either a fusion event between a bacterium and an archaeon that produced the first eukaryote or the direct evolution of the Eukarya from the Archaea. The Eukaryotes First proponents posit that the eukaryotes evolved initially and then, by reductive evolution, produced the Bacteria and Archaea. No mention was made of another previously published hypothesis termed the Nuclear Compartment Commonality (NuCom) hypothesis, which proposed the evolution of the Eukarya and Bacteria from nucleated ancestors (Staley 2013 *Astrobiol Outreach* **1**, 105 (doi:10.4172/2332-2519.1000105)). Evidence from two studies indicates that the nucleated Planctomycetes–Verrucomicrobia–Chlamydia superphylum members are the most ancient Bacteria known (Brochier & Philippe 2002 *Nature* **417**, 244 (doi:10.1038/417244a); Jun *et al.* 2010 *Proc. Natl Acad. Sci. USA* **107**, 133–138 (doi:10.1073/pnas.0913033107)). This review summarizes the evidence for the NuCom hypothesis and discusses how simple the NuCom hypothesis is in explaining eukaryote evolution relative to the other hypotheses. The philosophical importance of simplicity and its relationship to truth in hypotheses such as NuCom and Domain Cell Theory is presented. Domain Cell Theory is also proposed herein, which contends that each of the three cellular lineages of life, the Archaea, Bacteria and Eukarya domains, evolved independently, in support of the NuCom hypothesis. All other proposed hypotheses violate Domain Cell Theory because they posit the evolution of different cellular descendants from ancestral cellular types.

## 1. Introduction

Carl Woese used the small subunit rRNA to construct the scientific Tree of Life [1]. This phylogenetic tree provided evidence that life consists of three domains, the Bacteria, Archaea and Eukarya. The major question this review addresses is ‘What hypothesis best explains the evolution of the three domains, and in particular, the Eukarya?’

That the origin of the Eukarya is still a hotly debated subject is attested to by the contributions to a recent meeting of the Royal Society in London in 2015 [2]. Some participants did not commit to a hypothesis, but those who did fell into two primary camps. Most advocated a ‘Prokaryotes First’ hypothesis and one paper discussed the various ‘Eukaryotes First’ hypotheses.

Those who favoured ‘Prokaryotes First’ hypotheses trace their ideas most recently to the Ring Theory of Life [3]. A basic argument of the Prokaryotes First proponents is that, because prokaryotic (*before* nucleus) organisms are simpler and evolution leads to greater complexity, the prokaryotes, i.e. the Bacteria and Archaea, must have been the first organisms.

Mariscal & Doolittle [4] summarized a different set of hypotheses from scientists who favoured a ‘Eukaryotes First’ hypothesis. The major claim of these advocates is that the Eukarya must have evolved first to produce the Bacteria and Archaea because it is simpler to produce a prokaryote from a eukaryote by reductive evolution than vice versa.

Unfortunately, an entirely different hypothesis termed the Nuclear Compartment Commonality (NuCom) hypothesis [5] was not discussed at the meeting although it was published prior to the meeting. NuCom posits that the Bacteria and Eukarya evolved from nucleated ancestors. The Bacteria evolved from nucleated ancestors of the Planctomycetes–Verrucomicrobia–Chlamydia (PVC) superphylum. In addition, it posits that the Eukarya have always been nucleated. A major purpose of this paper is to briefly summarize and then provide a critique of the Prokaryotes First and Eukaryotes First hypotheses. This is followed by information about NuCom, a ‘Nucleated Organisms First’ hypothesis, because it is virtually unknown to biologists.

Finally, cell theory will be discussed. Current cell theory holds that every cell comes from a cell. Domain Cell Theory, proposed below, states that when the domains of life evolved, each of the three domains evolved from separate and unique cellular lineages.

## 2. Terminology

The *PVC Bacteria* are the PVC superphylum [6], some members of which are nucleated, i.e. their DNA and DNA replication, and probably also transcription, occur in a membrane bound compartment composed of glycerol 3-phosphate with *sn*-1,2 stereochemistry linked to the fatty acid side chains by ester bonds (G3P PLFA). Some species such as *Gemmata obscuriglobus* have cellular compartments with nuclei [7]. In addition to the PVC phyla, the phyla Lentisphaerae and Poribacteria may also be members of the PVC superphylum.

*Enucleation* is the process whereby a nucleated organism loses its nuclear compartment through reductive evolution. For example, nucleated ancestors of the Verrucomicrobia may have evolved to produce the Proteobacteria because both contain methanotrophic bacteria, use the Calvin–Benson carbon dioxide fixation process and contain prosthecate bacteria [5,8].

*Common bacteria* are defined as typical Bacteria, like *Escherichia coli*, a proteobacterium whose DNA is not contained in a nuclear compartment.

**Figure 1.**
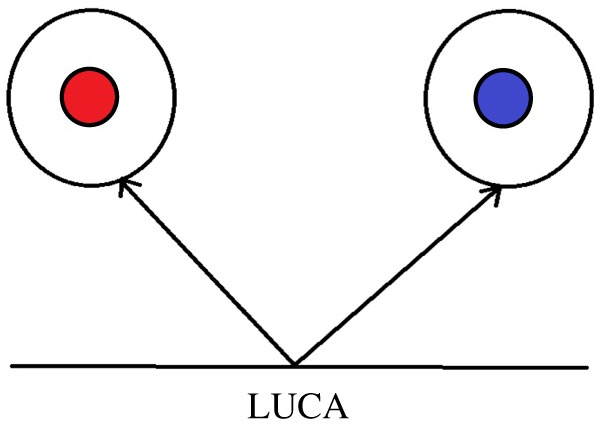
Illustration showing the evolution of the Bacteria and Eukarya from LUCA. The bounding cell and nuclear membranes of the Bacteria (red nucleus) and Eukarya (blue nucleus) have an essentially identical chemical composition, however the genomes contain divergent genetic material.

*Protokaryote* (Greek *proto* meaning ‘first’ and *karyon* ‘nucleus’) refers to the pre-domain ancestral cell state of the last universal common ancestor (LUCA). NuCom proposes that the ancestor of the PVC Bacteria and the Eukarya were two emergent phylogeneticaly distinct protokaryotic lineages with a simple nuclear compartment (figure 1).

*Protokaryotic signature proteins* (PSPs) are homologous proteins currently found as remnants in certain representatives of the PVC superphylum as well as almost all representatives of the Eukarya (some have termed these ‘eukaryotic signature proteins').

*Eukaryogenesis* is defined as the continuous evolution of eukaryote cellular complexity and organization in this unique domain.

## 3. Summary and critique of hypotheses

### 3.1. Prokaryotes First hypotheses

A popular view held by many is that the Archaea are the ancestors of the Eukarya either by evolving directly to produce the Eukarya [9] or via an unproven and untestable fusion event between an archaeon and a bacterium. This latter view is commonly held by most microbiologists who regard the Bacteria and Archaea as prokaryotes, which implies that they were the first organisms that later gave rise to the nucleated Eukarya via a hypothetical fusion event.

Advocates of Prokaryotes First hypotheses at the 2015 Royal Society meeting fell into two groups:

Group A: Fusion event occurred between a bacterium and an archaeon that led to the evolution of the Eukarya

Fusion advocates have invoked the synthesis of the eukaryotic cell from the biological merger between a bacterium with an archaeon. The most challenging issue facing the ‘Prokaryotes First’ fusion advocates is that they need to explain how two highly divergent cell types produced a protoeukaryote. This is difficult to explain from three primary standpoints.

Firstly, and perhaps most importantly, if fusion occurred it was a ‘once-only’ or singular event. This singular event cannot be reproduced in the laboratory or subjected to rigorous scientific study. From a philosophical standpoint, a hypothesis that is not verifiable or falsifiable is unscientific [10] and therefore invalid.

Secondly, several questions remain unanswered. For example, how did the resulting eukaryote retain one cell membrane type rather than another? Several groups question, for example, the ability of an archaeon to engulf a bacterium, a necessary step in the entrainment of a mitochondrion in eukaryotic evolution [11,12] and fusion hypotheses in general [13].

Thirdly, this hypothesis is in violation of Domain Cell Theory because one cellular lineage was created by the fusion of two different cell types (see below).

Group B: The Archaea were the direct ancestors of the Eukarya

Other ‘Prokaryotes First’ advocates propose that the Archaea, alone, evolved to produce the Eukarya [9]. This currently popular view has been favoured more recently especially because of environmental genomic studies such as the recent discovery of the ‘Lokiarchaeum’ group of Archaea, in which evidence for ‘complex eukaryotic genes' has been found in environmental genome libraries [14].

Williams & Embley [9] proposed a two domain tree of the Bacteria and Archaea in which the Archaea evolved to produce the Eukarya. Their tree can be seriously questioned from other scientific information. For example, how did the Eukarya acquire their G3P PLFA membranes from an archaeon whose ether-linked membranes are completely different [15]? In addition, Gribaldo *et al*. [16] raise doubt about sufficient evidence for a monophyletic lineage containing the Archaea/Eukarya.

Both groups of the Prokaryotes First school of thought have retained an early view of organism evolution that believes eukaryotes must have evolved from simpler organisms. There is virtually no evidence that this is what actually happened.

Also, some microbiologists proposed that the PVC superphylum is ancestral to the Eukarya. More recently, McInerny *et al*. [17] rebutted this hypothesis. The view that the PVC group evolved to become the Eukarya was also doubted by Staley *et al*. [18], who conducted the first genomic study of a member of the Verrucomicrobia (*Prosthecobacter dejongeii*) and a Planctomycete (*Gemmata* strain Wa1-1). That study concluded it was unlikely that the PVC superphylum gave rise to the Eukarya.

Although the latter reference agreed with most of the conclusions of McInerny *et al.*, this author believes they wrongly regarded the ‘ESPs’ (eukaryotic signature proteins), such as bacterial tubulin and serine threonine kinase (STK) genes as horizontal gene transfers (HGTs) from Eukarya [18,19]. By regarding these ancient proteins as being more recent transfers from eukaryotic organisms, they have denied the PVC bacteria of their ancient heritage as discussed below in a more recent paper supporting the NuCom hypothesis [19]. This last reference regards these ancient proteins as remnants from LUCA that were essential in the early evolution of the nucleated Bacteria and Eukarya. As such, they provide important phylogenetic evidence for the early commonality between the Bacteria and Eukarya.

Significantly, although most of these ancient proteins have been explained as more recent HGT events by some [17], the enzymes responsible for cell membrane synthesis are unlikely to be due to HGT primarily because membranes must have pre-dated the origin of cellular life [19]. Further, so far as is known, all the enzymes of the Bacteria and Eukarya that are responsible for the synthesis of G3P PLFA membranes are homologous [15], supporting the commonality of cell membranes in LUCA for the Bacteria and Eukarya, and the NuCom hypothesis. One might, though, also predict homologous membrane enzymes from Prokaryotes First hypotheses which propose the alpha-proteobacterium ancestor of the mitochondrion was engulfed by the Archaea host that evolved to produce the Eukarya. However, those hypotheses must explain how the mitochondrial G3P PLFA membrane replaced the ether-linked membrane of the host archaeon in view of arguments for Simplicity and the Cellular Compatibility (discussed later in this paper).

### 3.2. Eukaryotes First hypotheses

Hypotheses of the Eukaryotes First proponents were also presented at the Royal Society meeting [4], with various views of those who believe that the Bacteria and Archaea are descended from nucleated, eukaryotic organisms. However, as Woese's Tree of Life indicates, the Eukarya did not give rise to the Bacteria because they appear on a completely separate branch of the Tree of Life.

This author agrees with one very important point that the Eukaryotes First proponents espouse, namely that it is simpler to produce a prokaryote from a eukaryote (i.e. a nucleated organism) than to produce a eukaryote from one or two prokaryotes. This point of view is consistent with the NuCom hypothesis that explains the evolution of the Bacteria and Eukarya from nucleated ancestors.

## 4. Nuclear Compartment Commonality hypothesis

The NuCom hypothesis [5,19] states that both the Eukarya and the Bacteria evolved from nucleated ancestors during the period that DNA replication evolved. This is in agreement with the view that the Eukarya comprise an independent domain and have always been nucleated [20]. A more precise timetable regarding this is not possible at this time without additional information, but the section Eukaryogenesis below states that it may have occurred about 3.0 Ga bp.

According to NuCom, the Bacteria are also descended from nucleated organisms. Phylogenetic evidence supporting NuCom comes from two independent groups. One group provided phylogenetic information from highly conserved regions of 16S rDNA [21] that indicates the Planctomycetes are the most ancient Bacteria. Likewise, Jun *et al*. [22] arrived at the same conclusion using proteomic phylogenies. Ancestors of the nucleated PVC superphylum are hypothesized by NuCom to be ancestral to all other Bacteria including the enucleate Common Bacteria.

The Common Bacteria are regarded as having become enucleate by reductive evolution from PVC superphylum ancestors. The rationale for this is that by maintaining a smaller and less complex genome, they could compete more efficiently for their niches. The example given in the original NuCom hypothesis is that of the Verrucomicrobia giving rise to the Proteobacteria [5]. Both groups contain the only methanotrophic members of the Bacteria and share other features as well, such as prosthecae and the Calvin–Benson cycle. Most importantly, a 16S rRNA phylogenetic tree supports the view that the Verrucomicrobia were the ancestors of the Proteobacteria [8].

Further phylogenetic support for NuCom comes from ancient PSPs such as α- and β-homologues of tubulin that have been found in the PVC superphylum. These proteins have been called ESPs by many because they are found in phylogenetic trees with eukaryote homologues. By contrast, these proteins, which are found in a few representatives of the PVC superphylum, are regarded as PSPs of LUCA by the NuCom hypothesis because of their ancient phylogeny. They occur as remnants in certain species indicating that reductive evolution has occurred in PVC phyla as well as in the enucleate Common Bacteria but their presence in the PVC reveals their deep ancestry from LUCA.

Finally, and perhaps most importantly, the NuCom hypothesis is the simplest hypothesis to explain the origin of the Eukarya. The Simplicity analysis, which regards the simplest hypotheses and theories to be more likely true philosophically, is used in physics and in chemistry, although much less often in biology. Hypotheses and theories that are the most simple are considered not only most likely to be true, but are also aesthetically more favourable [23]. This argument applies to both the NuCom hypothesis and Domain Cell Theory because they require much less complexity to explain the evolution of the Bacteria and Eukarya.

## 5. Eukaryogenesis

Prokaryotes First advocates believe that the Eukarya arose later in time because fossil evidence for them is non-existent until about 1.5–2.0 Ga bp. However, NuCom regards that eukaryotes evolved during the time that DNA replication evolved in the PVC Bacteria. The NuCom hypothesis explains the later appearance of the Eukarya in the fossil record by a series of stages of a long process of complexification termed eukaryogenesis (dates below are approximate).

Stage A. Evolution of DNA replication in LUCA.

When DNA replication evolved in LUCA it gave rise to two disparate lineages, the Bacteria and the Eukarya. Therefore, both lineages date to about 3.0 Ga bp.

*Stage B. Early period for the Eukarya: detection of Eukarya was difficult because:*

(1) Cells were unicellular with only a membrane enveloping them—therefore they did not leave identifiable fossil traces.
(2) Few cells were formed because they had a poor energy source—they probably were fermentative and lived off available sugars.
(3) No exceptional, unique products were produced by eukaryal metabolism—unlike methanogenic Archaea which give rise to ^12^C-fractionated methane or Bacteria such as the Cyanobacteria that produced oxygen.
(4) Actin evolution began about 2.5 Ga bp. This led to the ability of Eukarya to engulf foodstuffs, the singular early means that still characterizes the unique eukaryal feeding mode, phagocytosis. Notably, we as human omnivores still use it and make a big fuss about it, too!

*Stage C. Mitochondrial entrainment—ca 2.5 to 2.0 Ga bp (all known Eukarya either have mitochondria or are descended from organisms that had them*).

(1) The mitochondrion evolved from an aerobic member of the Alphaproteobacteria which, after engulfment, was entrained by symbiosis within the ancestor of all Eukarya. This enormously enhanced their ability to make ATP.
(2) These early Eukarya were still unicellular and difficult to detect because they lacked cell walls.
(3) The Cellular Compatibility argument (see below) provides a rationale for how a bacterium became the mitochondrion.

*Stage D. Period of evolution of mitosis, meiosis and sexuality and larger, more complex multicellular organisms. About 2.0–l.5* *Ga bp until the present*.

Eukaryogenesis occurred over many millions of years, but it was not until they had fully evolved that the Eukarya as we know them today could be readily detected in the fossil record.

Although the early stages (A – early stage D) could not have been easily detected in the fossil record, by about 1.5–2.0 Ga bp, the evolution of the Eukarya eventually gave rise to the more readily detectable contemporary single and multicellular organisms including certain protists, algae, plants and animals.

## 6. Homlogous proteins found in Planctomycetes–Verrucomicrobia–Chlamydia Bacteria and Eukarya

Several examples of ancient highly conserved proteins (PSPs) are found in the PVC superphylum as well as the Eukarya. A summary of this information is provided below that supports the common origin of these proteins in LUCA and the nucleated descendants of the PVC Bacteria and Eukarya.

### 6.1. Cell membrane enzyme homologues

As noted previously, the cell membranes (and hence nuclear membranes of the nucleated organisms) of the Bacteria and Eukarya are identical so far as is known. They both comprise G3P PLFA. The pathway for their synthesis is also identical so far as is known, including homologous enzymes for each of the steps [15]. This is *prima facie* evidence for the ancient common ancestry of these PSPs in both Bacteria and Eukarya completely in accord with NuCom.

Some have proposed these cell membrane proteins (enzymes) represent HGTs between the Bacteria and Eukarya. For example, in their review Poole & Penny [24] discuss one unlikely scenario that suggests the eukaryotic membrane may have been derived from the bacterial mitochondrial endosymbiont of the purported archaeon ancestor as discussed previously.

Interestingly, although cell and nuclear membranes represent early commonalities between the Bacteria and Eukarya, their genomes diverged from one another enormously and gave rise to the two most plentiful and diverse forms of life on Earth.

Most importantly, these cell membrane proteins are found in *all* Bacteria, not simply the PVC superphylum. These ancient membrane proteins from LUCA provide irrevocable testimony to a common origin of the Bacteria and the Eukarya that is consistent with NuCom.

The following ancient PSPs found in the PVC superphylum have been regarded by others as due to HGT from a eukaryote. However, there is no basis for this other than that they are found in phylogenetic trees close to those of the Eukarya. NuCom rightly reclaims them as PSPs derived from LUCA and not examples of HGT events.

### 6.2. Tubulin

Perhaps the most remarkable protein reported in the PVC superphylum is tubulin. The α- and β-homologues of tubulin have been found in all members of the Eukarya. Lynn Margulis, an early proponent of Prokaryotes First concepts, hypothesized that tubulins came from the spirochetes, which was consistent with her view that the spirochetes evolved tubulin that provided motility to some immotile protists. However, no spirochete genome has ever been reported to contain tubulin genes whereas some of the Verrocomicrobia do. Several species of the *Prosthecobacter* genus contain the highly conserved tubulin proteins bacterial tubulin A (BtubA) and bacterial tubulin B (BtubB) which are homologous to α- and β- eukaryotic tubulin, respectively [25].

FtsZ is a much smaller homologue of tubulin that is found in all Bacteria and some of the Archaea and is required for cell division. Importantly, FtsZ is also found in the tubulinate *Prosthecobacter* species [19], suggesting it is necessary for cell division. Aside from these bacteria, no other known species of the Bacteria or the Archaea is known to have tubulin homologues.

### 6.3. Ubiquitin system and serine/threonine kinases

The ubiquitin system contains enzymes that are responsible for the degradation of proteins and is found in all eukaryotes. Eukaryote-like serine/threonine kinases (STKs) and E2-ubiquitin-conjugating enzymes are also found in the PVC superphylum, including members of the Planctomycetes [26], Chlamydia and Verrucomicrobia.

### 6.4. Sterol synthesis

Some members of the Planctomycetes contain sterols in their cell membranes which are also found in the Eukarya as well as some Common Bacteria. Importantly, the sterol synthesis pathway in the *Gemmata* genus contains deeply rooted enzymes (PSPs) consistent with their origin in LUCA [19], although some have inferred they are another example of HGT between the Eukarya and Bacteria [27]. The extensive pathway network found in *Pirellula staleyi* provides strong support for the pathway in extant members of the PVC superphylum [19].

## 7. Other support for Nuclear Compartment Commonality

Studies of the phylogeny of ancient protein folding families (FF) are also consistent with NuCom [28]. These authors report that early evolution progressed in five phases as shown in Venn diagrams. The initial phase indicated there were 76 shared FF among all three domains. The final Phase V contained 484 FF shared among the three domains. At stage V, however, the total number of protein FF found in the Archaea was only 703, whereas there were 1510 in the Bacteria and 1656 in the Eukarya. Further, this paper indicates that the Archaea branched off LUCA with these fewer FF compared with the Bacteria and Eukarya, which remained together before later diverging dramatically as sister groups, is in accord with Woese's Tree of Life.

This perspective article also proposes the Domain Cell Theory of Life which supports the NuCom hypothesis because Woese's three domains of life comprise three independent cellular lineages. Fusion between two cellular types does not occur. The entrainment of an alpha-proteobacterium to become a mitochondrion and a cyanobacterium to become a chloroplast in the Eukarya do not change the fundamental cellular type of the Eukarya in which they became endosymbionts.

## 8. Domain Cell Theory of Life

Schleiden and Schwann proposed the cell theory of life which states that all living organisms are cellular. All cells are derived from pre-existing cells. As stated by Virchow in 1859 [29], ‘every cell from a cell’ (*omnis cellula e cellula*).

The Tree of Life provides a further elaboration of the meaning of cell theory. This is herein named Domain Cell Theory, which posits that the domains in Carl Woese's Tree of Life comprise three *different cellular types*: Archaea, Bacteria and Eukarya.

Domain Cell Theory (all organisms are cellular) contains the following tenets:

1. Only Bacteria can give rise to other Bacteria with their unique genetic composition and evolutionary trajectory.
  — All Bacteria have cell membranes containing glycerol 3-phosphate with *sn*-1,2 stereochemistry linked to the fatty acid side chains by ester bonds (G3P PLFA).
  — All Bacteria have or have had peptidoglycan cell walls during their evolution.
  — The PVC superphylum contains the most ancient members of the Bacteria, some of which are nucleated.
2. Only Eukarya can give rise to other Eukarya with their unique genetic composition and evolutionary trajectory.
  — All Eukarya have cell membranes containing glycerol 3-phosphate with *sn*-1,2 stereochemistry linked to the fatty acid side chains by ester bonds (G3P PLFA).
  — All members of the Eukarya contain a nucleus with nuclear membranes.
  — Eukaryogenesis describes the process by which Eukaryotic cells evolved engulfment of particulate materials (phagocytosis), mitosis, meiosis and sexuality.
3. Only Archaea can give rise to other Archaea with their unique genetic composition and evolutionary trajectory.
  — All Archaea have glycerol 1-phosphate (G1P) ether linkages in their cell membranes.

In particular, each of the three cellular lineages is distinct from the other two on the basis of cellular evolution, genetic composition and cell envelope types, which include cell and nuclear membrane and cell wall, if present. This is logical because cell membranes must have existed at the time LUCA gave rise to the three separate domain lineages. Domain Cell Theory states that the descendants of each of the three domains retained its identity throughout its own unique evolutionary pathway.

A primary feature or tenet of Domain Cell Theory is that it is not possible to produce a different cell type from the fusion of two other cellular types as proposed by ‘Prokaryotes First’ proponents. Another characteristic is that it is impossible for one cellular type to become a different cellular type, such as the direct evolution of the Eukarya from the Archaea [9]. Accordingly, all ‘Prokaryotes First’ hypotheses are invalid by Domain Cell Theory which is in agreement with the NuCom hypothesis.

Likewise, the various ‘Eukaryotes First’ proposals that the Archaea and Bacteria evolved from the Eukarya are also contrary to the Tree of Life, which clearly indicates a separate evolution of all three cellular lineages. In particular, the Eukaryotes First proponents propose that the Eukarya evolved by reductive evolution to produce the Bacteria and Archaea. This is also a violation of Domain Cell Theory. Thus, none of the Prokaryotes First or the Eukaryotes First hypotheses is valid.

Further support for Domain Cell Theory is that, of all the thousands of Bacteria, Archaea and Eukarya that have been studied, each of them can be placed into one of the three domains of life. If cellular types are freely able to change, then one should ask, ‘Where are the intermediate types or species among these three different domains?’ To my knowledge, none exist.

Regarding Domain Cell Theory discussed above, two mitigating factors need to be mentioned. The first is that viruses are known to play a very important role in transferring genes from one organism to another, which could lead to the introduction of genes into different lineages. An especially interesting proposal is that the three domains of life originated in an RNA world in which, during the transition to DNA, three different founder DNA viruses gave rise to the three domains of organisms [30].

In addition, HGT is also known to be a mechanism to transfer genes from one group of organisms to another. However, it is noteworthy that these events, including viral transfer, typically occur between closely related taxa. Unfortunately, HGT has been sometimes misused to support hypotheses that are otherwise unsupportable.

## 9. Cellular Compatibility argument

It is interesting to note that modifications of cells do occur within at least two of the domains, the Bacteria and the Eukarya. Regarding the Bacteria, according to NuCom, the original nucleated ancestors of the PVC superphylum gave rise to the enucleate Common Bacteria which lost their nuclei through reductive evolution [5]. This probably occurred to enable Common Bacteria to fit more efficiently into their specialized niches that required less energy. This process does not violate cell theory in that the cell envelope of all Bacteria still contains peptidoglycan as well as the G3P PLFA membranes. Earlier it was thought that the Planctomycetes lacked peptidoglycan, but more recently it has been found in two different members of the phylum [31,32], indicating that all bacteria contain it except for those such as bacterial L-forms that have lost it.

With respect to the Eukarya, all species have a mitochondrion or did have a mitochondrion during their evolution. Those that no longer have mitochondria lost them through reductive evolution. This is explained by Cellular Compatibility as follows. The proto-eukaryotic cells lacked mitochondria because the Alphaproteobacteria lineage that gave rise to the mitochondrion had not evolved until approximately 2 Ga bp. Because cell membranes of the Bacteria and Eukarya are highly similar, the two cellular types are compatible. The engulfed pre-mitochondrion lost its peptidoglycan layer during the entrainment process making it compatible within eukaryotic cells. A similar argument can be made for chloroplasts that are derived from Cyanobacteria that were entrained in algae following ingestion by single-celled protists enabling them to become photosynthetic. Interestingly, these entrained cells also lost their peptidoglycan. The plants that also have cell walls evolved from the algae with cyanobacterial chloroplasts.

By contrast, there is no evidence that the Archaea produced intracellular organelles of or in the Bacteria or Eukarya. Cellular incompatibility may account for this because the archaeal cell envelopes are markedly different from the Bacteria or Eukarya. Although the evolution of the Archaea is not discussed here, their evolution was independent from that of the Bacteria and Eukarya because of their unique cell envelope features and generally smaller genomes. This is in agreement with the Tree of Life and the NuCom hypothesis.

## 10. Discussion

Carl Woese's Tree of Life was a seminal event in biology because it was the first scientific Tree of all organisms on Earth. His Tree of Life actually revealed that the Archaea comprise a separate, third branch of life that was previously unknown. What is so surprising is that the interpretation of the Tree of Life has been so controversial.

Three main lines of thought have contributed to the confusion about what the Tree of Life means:

Life evolved from prokaryotic organisms, the Bacteria and Archaea, because they are simpler than the Eukarya and evolution leads to greater complexity

Students of microbiology are taught about prokaryotic organisms. Since ‘pro’ means ‘before’ this implies that the Bacteria and Archaea were the first organisms, i.e. prior to the Eukarya. Secondly, many regard evolution as a process that always leads to complexity, so simple organisms, i.e. prokaryotes, probably evolved to produce nucleated organisms. Proponents of ‘Prokaryotes First’ hypotheses frequently believe that the Bacteria and Archaea contain all the genetic information necessary to produce a eukaryotic organism.

All members of the Bacteria including the PVC superphylum are prokaryotic

Although members of the PVC superphylum contain nuclei, many microbiologists either do not know this or they expect these cannot be nuclei because they are not identical to those of the Eukarya despite the marked divergence of the Bacteria from the Eukarya as shown by the Tree of Life. They then further deny the PVC superphylum of their remarkable phylogenetic characteristics such as the PSP proteins which are among the most complex and significant phylogenetic markers confirming their ancient heritage in LUCA. Their explanation for their occurrence in the PVC superphylum is, without evidence, that they have been transferred by HGT from the Eukarya.

*The Eukarya did not evolve until about 1.5–2.0* *Ga bp*

Because the Eukarya did not leave a fossil record until about 1.5 Ga bp, many believe it was because they evolved more recently than the Bacteria and Archaea. This view also promotes the idea of Prokaryotes First hypotheses.

As discussed here, this author believes that all three of these views are misconceptions that have led to incorrect interpretations of the meaning of the Tree of Life. A simple examination of Woese's Tree shows there are three major, independent lines of descent, the Bacteria, Eukarya and Archaea, leading to the concept of three domains. Moreover, the late appearance of the Eukarya which is clearly indicated by the long branch of this lineage in Woese's Tree of Life is not because they evolved last, but because their complete evolution required much more time and they did not leave detectable evidence in the fossil record until much later. Present-day eukaryotes required hundreds of millions of years of evolution to attain the hallmarks of the present-day Eukarya: complex genomes with mitochondria that could carry out mitosis and meiosis, sexuality and could develop noticeable traces of their existence in the fossil record.

Of course those who believe that the Eukarya evolved first do not accept the Prokaryotes First interpretation either. However, their idea that the Eukarya evolved to produce the Bacteria and Archaea does not fit with Woese's Tree either because each of life's domains is independent from the others.

This paper also posits the Domain Cell Theory, which is an extension of Cell Theory. As clearly shown in Carl Woese's Tree, there are three independent cellular lineages of life. The product of each led to a descendant cell type of its own kind: Bacteria from Bacteria, Archaea from Archaea and Eukarya from Eukarya.

The NuCom hypothesis is the only one that complies with Domain Cell Theory. All Prokaryotes First and Eukaryotes First hypotheses violate Domain Cell Theory.

## Data accessibility

This article has no additional data.
